# Advances in small molecule two-photon fluorescent trackers for lipid droplets in live sample imaging

**DOI:** 10.3389/fchem.2022.1072143

**Published:** 2022-11-25

**Authors:** Dong Joon Lee, Eun Seo Kim, Hyo Won Lee, Hwan Myung Kim

**Affiliations:** ^1^ Department of Energy Systems Research, Ajou University, Suwon, South Korea; ^2^ Research Institute of Basic Sciences, Suwon, South Korea; ^3^ Department of Chemistry, Ajou University, Suwon, South Korea

**Keywords:** lipid droplet, lipid metabolism, organelle tracker, two-photon microscopy, live sample imaging

## Abstract

Two-photon fluorescent trackers for monitoring of lipid droplets (LDs) would be highly effective for illustrating the critical roles of LDs in live cells or tissues. Although a number of one-photon fluorescent trackers for labeling LDs have been developed, their usability remains constrained in live sample imaging due to photo damage, shallow imaging depth, and auto-fluorescence. Recently, some two-photon fluorescent trackers for LDs have been developed to overcome these limitations. In this mini-review article, the advances in two-photon fluorescent trackers for monitoring of LDs are summarized. We summarize the chemical structures, two-photon properties, live sample imaging, and biomedical applications of the most recent representative two-photon fluorescent trackers for LDs. Additionally, the current challenges and future research trends for the two-photon fluorescent trackers of LDs are discussed.

## Introduction

Lipid droplets (LDs) are the key organelles in live cells that mainly serve as the lipid repository containing neutral lipids, including triacylglycerol (TAG) and cholesterol ester (CE) (Murphy et al., 1999; Tauchi-Sato et al., 2002; Martin and Parton, 2005; Welte and Gould, 2017). LDs are related to many aspects of cellular biology processes, such as lipid transport, energy storage, signal transduction, and cellular homeostasis (Martin and Parton, 2005; Farese and Walther, 2009; Zehmer et al., 2009; Blom et al., 2011; Greenberg et al., 2011; Olzmann et al., 2013). In recent years, LDs have been prominently regarded as biomarkers of a variety of diseases, including fatty liver, immune dysfunction, obesity, and other diseases related to lipid regulation (Bozza and Viola, 2010; Herker et al., 2010; Krahmer et al., 2013; Abramczyk et al., 2015; Liu et al., 2017; Olzmann and Carvalho, 2019). Furthermore, LDs interact dynamically with other cellular organelles, such as endoplasmic reticulum (ER), plasma membrane, lysosomes, and mitochondria, mainly for LD formation, lipid homeostasis, autophagy, and lipolysis, respectively (Fujimoto and Parton, 2011; Olzmann and Carvalho, 2019; Dejgaard and Presley, 2021; Wang et al., 2021). Therefore, it is highly desirable to establish an imaging method to specifically label the LDs in live samples.

Several approaches have been performed to study the dynamics of LDs using imaging methods, including Raman scattering microscopy, electron microscopy, and atomic force microscopy (Fujimoto et al., 2013; Shoham et al., 2014; Zhang and Boppart, 2020; Chen et al., 2022). In particular, the fluorescence microscopy imaging method using LDs-specific fluorescent trackers is advantageous because of its convenience, non-invasiveness, and high sensitivity (Suzuki et al., 2014; Kim and Cho, 2015). To date, a number of LDs-selective fluorescent trackers have been developed (Wang et al., 2016; Collot et al., 2018; Zhang et al., 2020; Fan et al., 2021). These trackers have been utilized to label LDs in cells and tissues. However, the most commonly used commercial trackers for LDs are BODIPY 493/503 and Nile Red, which have low LD specificity (Zhao et al., 2022). Moreover, the one-photon (OP) excited fluorescence (OPEF) trackers, which are only applicable to the conventional one-photon microscopy (OPM) are restricted in live sample imaging due to the light scattering, autofluorescence, photodamage, and shallow imaging depth (Kim and Cho, 2009; Lee et al., 2014; Juvekar et al., 2022).

Two-photon (TP) microscopy (TPM), which utilizes two near-infrared (NIR) photons as the excitation source, has emerged as an advanced imaging tool for biomedical research, can afford outstanding features such as low self-absorption and background noise, high resolution, and photostability (Kim and Cho, 2015). TPM can also provide long-term, in-depth real-time imaging of live samples (Kobat et al., 2011; Wang et al., 2020b; Lozano-Torres et al., 2021). Moreover, LD dynamics are sensitive and might be perturbed by the exogenous fluorescent trackers to label them in live samples. It is reported that small molecular size, high target specificity, and low concentration of the fluorescent trackers could minimize such perturbation (Kowada et al., 2015; Werther et al., 2021; Li et al., 2022). However, the lack of small molecule LDs-trackers that are applicable for TPM limits the progress in the study of the dynamics of LDs. In this regard, the development of small molecule and high LDs-specific TP trackers optimized in the micromolar range and with a short incubation time is critical. Most recently, some small molecule TP trackers for LDs have been reported, and their excellent imaging capabilities to monitor the dynamics of LDs in live samples have been evaluated.

**FIGURE 1:**
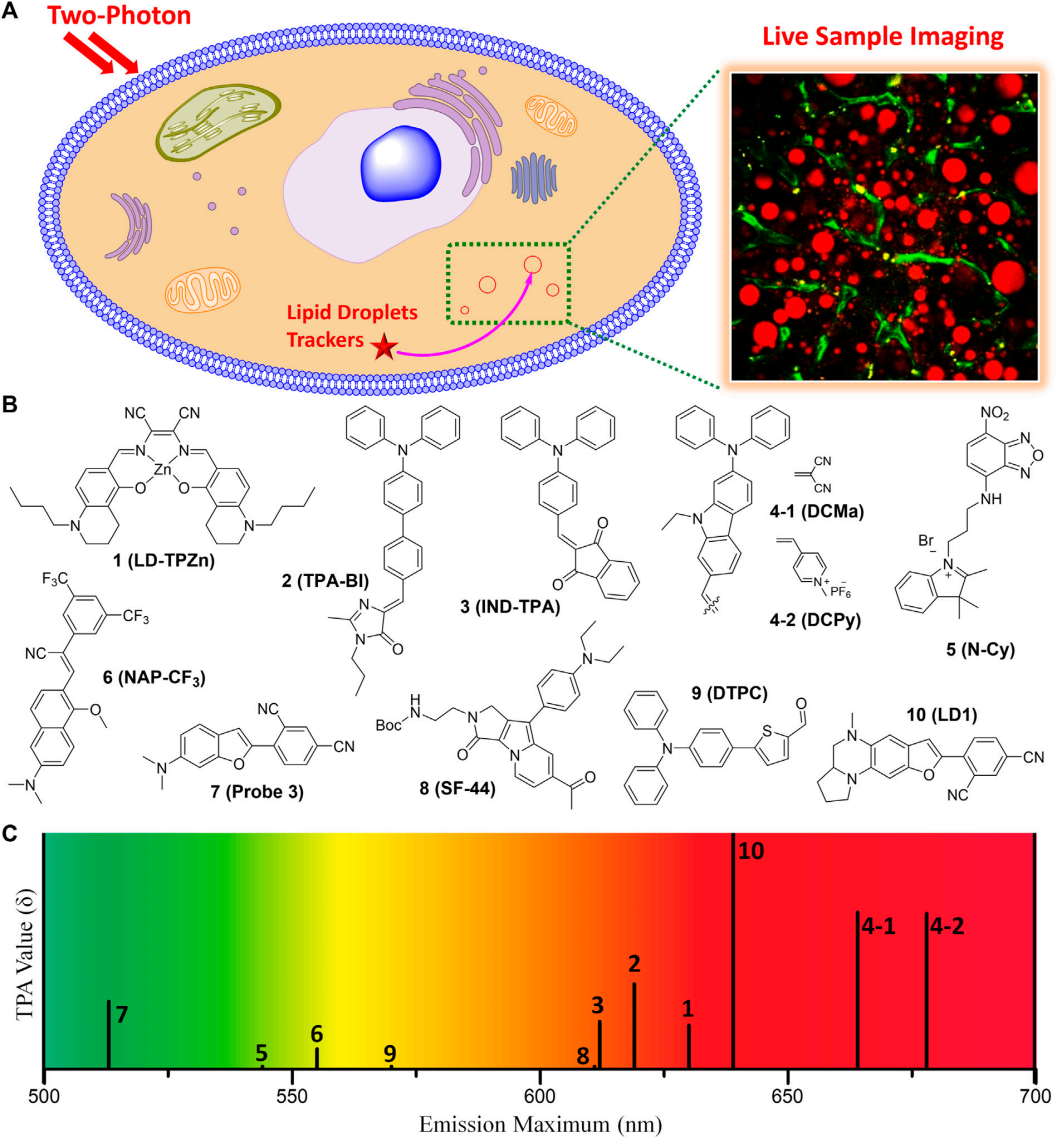
**(A)** Illustration for monitoring the dynamics of LDs in live sample by TPM imaging using TP fluorescent tracker. **(B)** Chemical structures of the representative small molecule TP trackers for LDs. **(C)** Schematic plot for the emission maxima and TPA values of **1**–**10**.

**FIGURE 2:**
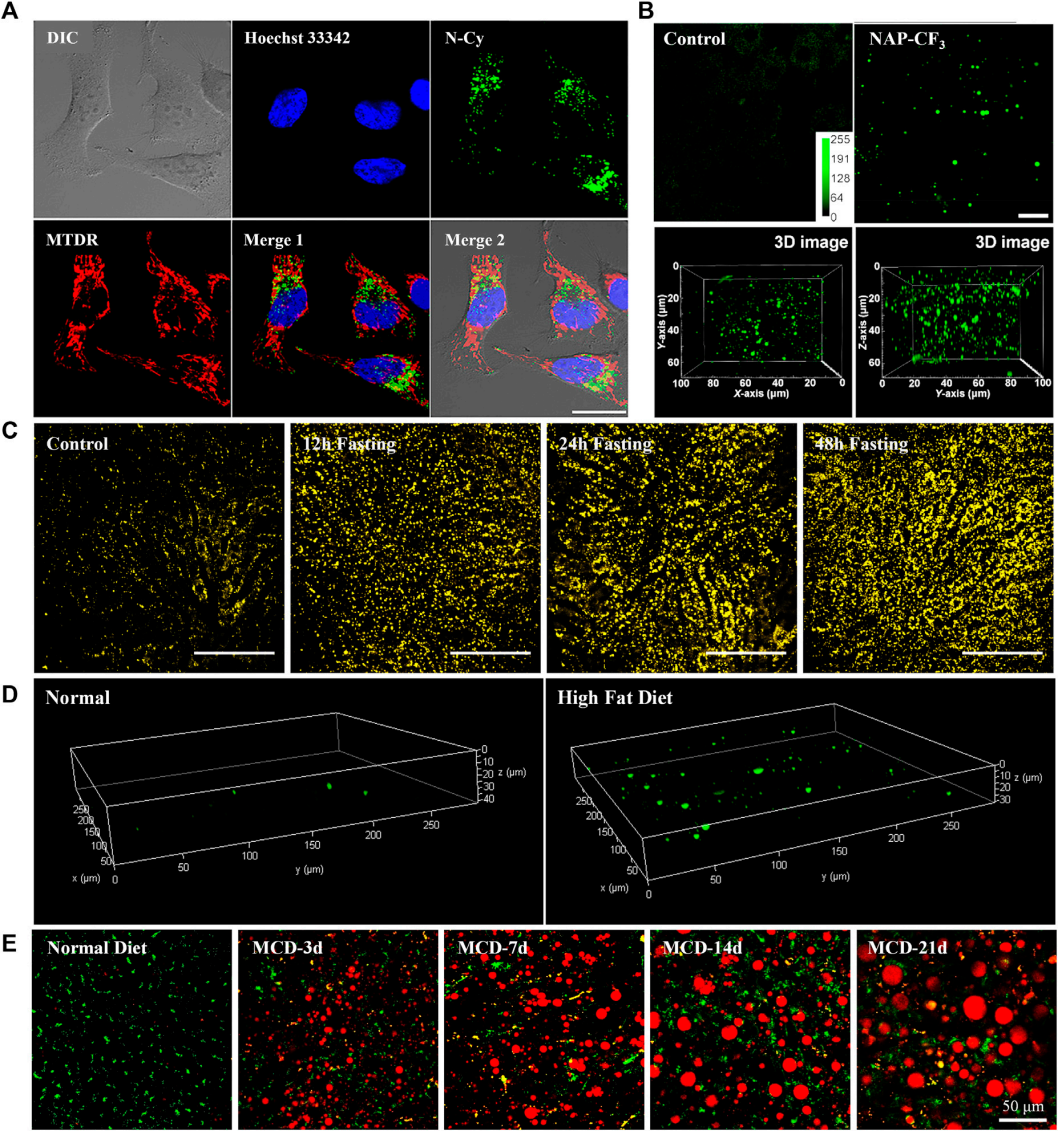
**(A)** Co-localization experiments of **5** (Green, LDs), Hoechst 33,342 (Blue, nucleus), and Mito Tracker Deep Red (Red, mitochondria). Reproduced with permission from Guo et al., 2018. Copyright 2018 American Chemical Society. **(B)** *Ex vivo* two-photon images of live mice liver tissue incubated with or without **6**, and the reconstructed 3D image. Reproduced with permission from Niu et al., 2018. Copyright 2018 American Chemical Society. **(C)** Representative images of hepatic LDs using **8** in the liver of anesthetized mice after normal feeding or fasting. Reproduced with permission from Moon and Kim, 2021. Copyright 2021 The Korean Society of Lipid and Atherosclerosis. **(D)** Reconstructed 3D TPM images of normal mouse liver tissue and fatty liver disease mouse tissue stained with **9**. Reproduced with permission from Han et al., 2022. Copyright 2022 Royal Society of Chemistry. **(E)** Merged images of dual-color TPM experiments of LDs (Red, **10**) and lysosomes (Green, BLT) in the liver of normal mice and MCD diets mice for various time periods. Reproduced with permission from Lee et al., 2022. Copyright 2022 American Chemical Society.

This mini-review briefly provides characteristics of the recent small molecule TP fluorescent trackers for LDs (Table 1). The chemical structures of the representative TP trackers are shown in Figure 1. Most of these trackers exhibit excellent two-photon excited fluorescence (TPEF) properties with LDs-specific labeling capabilities in live samples. The most impressive TP imaging experiments are shown in Figure 2.

**TABLE 1:** Photophysical data of the representative small-molecule TP LDs trackers.

Tracker	λ_abs_ ^a^	λ_fl_ ^b^	Φ^c^	Φδ^d^	δ^e^	[Tracker]^f^	Ex.^g^	Application
**1**	599	630	0.44	48	110	2.0	543/790	Rat adipose tissue
**2**	447	619	0.22	47	213	5.0	442/840	Mouse liver tissue
**3**	478–502	594–612	0.05–0.10	12	119	5.0	473/920	HCC827 cells
**4-1**, **4-2**	478, 454	664, 678	0.296, 0.07	117, 27	394, 390	1.0	488/900	Mouse liver tissue
**5**	518–535	524–544	0.004–0.30	N.A.^h^	N.A.	4.0	473/840	Mouse liver tissue
**6**	425	555	0.29	15	50	1.0	405/860	Mouse liver tissue
**7**	444	513	0.98	166	169	1.0	488/940	Huh-7 cells
**8**	445	611	0.20	N.A.	N.A.	5.0	465/900	*In vivo* mouse liver tissue
**9**	402	476–570	N.A.	73	N.A.	3.0	405/760	Mouse liver tissue
**10**	478–499	521–639	0.007–1.00	610	610	1.0	488/800	*In vivo* mouse liver tissue

^a^ Maximum wavelength of one-photon absorption in nm.

^b^ Maximum wavelength of one-photon emission in nm.

^c^ Fluorescence quantum yield.

^d^ Two-photon action cross-sections in GM (10^–50^ cm^4^ s/photon).

^e^ Two-photon absorption cross-sections in GM (10^–50^ cm^4^ s/photon).

^f^ Cell loading concentration in μM.

^g^ One/Two photon excitation wavelengths for the fluorescence microscopy imaging in nm.

^h^ Data not available.

## Characteristics and TP imaging applications of the representative TP LDs trackers

Most of the fluorescent trackers for LDs are characterized by small size, high lipophilicity, low amphiphilicity, and low conjugated bond number (Thiele and Spandl, 2008; Stockert et al., 2010). The fluorescence quantum yield (Φ) of the LDs trackers should be higher in non-polar solvents (especially in lipid-like environments) than in polar solvents. These can increase the specific labeling ability of the trackers to LDs. Nowadays, researchers have designed TP LDs trackers considering these properties, including strong electron push−pull dipole molecules that have efficient intramolecular charge transfer (ICT). Typically, these small dipole trackers consist of highly lipophilic moieties, strong electron donating groups combined with strong electron withdrawing groups. These features would offer enhanced two-photon absorption cross-section (TPA, δ) values, particularly in non-polar solvents, increasing the specific properties for LDs (Zhao et al., 2022). With the high LDs specificity and with considerable TPA values, we can monitor the dynamics of LDs in live samples *via* TPM imaging using the innovative TP LDs trackers (Figure 1A).

In 2017, Tang et al. introduced a Zn-Salen ligand complex TP LDs tracker (**1**, LD-TPZn, Figure 1B) and demonstrated its live cell imaging ability to specifically label LDs (Tang et al., 2017). **1** showed absorption maxima at 599 nm and emission maxima at 630 nm, with a fluorescence quantum yield of 0.44. Additionally, it exhibited a TPA of 110 GM in the 790–880 nm emission window. It showed high cell viability and photostability, compared with the commercial LDs trackers. The cellular uptake *via* clathrin-mediated endocytosis might contribute to the high LD specificity of **1**. Owing to the low light scattering, high resolution, and deep penetration features of the TPM, they successfully visualized the biogenesis of LDs in adipocytes and the 3D distribution of LDs in rat adipose tissues. Furthermore, **1** can be utilized as a TP LDs tracker to apply in adipose tissue, one of the difficult samples for conventional OPM imaging due to the severe light scattering.

Jiang and co-workers reported an aggregation-induced emission (AIE) based TP LDs tracker (**2**, TPA-BI, Figure 1B) and applied it to mouse liver tissue slices to target LDs (Jiang et al., 2017). This tracker is composed of a triphenylamine electron donor and a benzylidene imidazolone electron acceptor. AIE based trackers for LDs have many advantages of larger Stokes shift, higher brightness, and better photostability than commercial trackers. **2** displayed both twisted intramolecular charge transfer (TICT) and AIE characteristics with a considerable TPA of 213 GM. In live HeLa, HepG-2, and A549 cell lines, **2** showed bright OPEF and TPEF specifically in LDs, and was also applicable to LDs analysis by flow cytometry. Compared with the commercial LDs trackers, **2** was more appropriate for TPM imaging of LDs’ deeper penetration in tissues and it can be further applied to monitor the localized polarity of complex samples under TP excitation. Owing to the synthetic convenience, further modifications of this tracker for labeling of other organelles are feasible for various biomedical research and clinical diagnosis.

In the same year, Tang group synthesized 2-(4-(diphenylamino)benzylidene)-1H-indene-1,3(2H)-dione (**3**, IND-TPA, Figure 1B) as an AIE fluorescent tracker for LDs imaging (Gao et al., 2017). This tracker has both AIE and TICT properties; 478–502 nm absorption maxima; 594–612 nm emission maxima; and a fluorescence quantum yield of 0.07–0.10, dependent on the polarity. In live HCC827 and A549 cell lines, **3** exhibited high overlap with the commercial LDs tracker, fast cell loading ability, low cytotoxicity, and excellent TPA of 119 GM at 920 nm TP excitation. This easily accessible AIE tracker for specific imaging of LDs in live cells can be utilized to detect the dynamic change of LDs with high photostability and spatiotemporal resolution in the aggregate state. It is expected that **3** can serve as an efficient and accessible imaging tool for the study of biological functions of LDs in real-time.

In 2018, Zheng et al. reported NIR AIE based TP trackers (**4-1** and **4-2**, **DCMa** and **DCPy**, Figure 1B) and demonstrated their application to photodynamic therapy (PDT) of cancer cells (Zheng et al., 2018). They have synthesized four compounds containing a triphenylamine donor with twisted molecular conformations exhibiting AIE characteristics. Among these, **4-1** and **4-2** include malononitrile and methylpyridinium salt, respectively, as an electron withdrawing group, and exhibited a high TPA of about 390 GM at 900–940 nm TP excitation. Though the conjugation length of **4-1** is similar to that of **2**, **4-1** showed 45 nm bathochromic emission shift and about 2-fold TPA increase compared to **2**. These might be due to the planar configuration of the carbazole linker of **4-2** instead of the rotatable diphenyl linker of **2**. With a very low imaging concentration of 1.0 μM, **4-1** can efficiently track LDs in live samples with a tissue penetration depth of 150 μm. On the other hand, **4-2** specifically stained mitochondria, probably due to its cationic character. Importantly, **4-2** efficiently generated singlet oxygen under white-light irradiation, allowing its useful application in the PDT of cancer cells. **4-2** has great potential as the OP and TP imaging agent for organelle-specific and image-guided PDT of cancer. Overall, **4-1** and **4-2** not only serve as NIR emission, efficient TPA properties, and organelle-specific labeling ability, but also may facilitate the development of TP imaging agents for organelle-specific therapy.

Yu group utilized an amphiphilic TP LDs tracker (**5**, **N-Cy**, Figure 1B) by introducing a 2,3,3-trimethyl-indoleninium group into a nitrobenzoxadiazole fluorophore (Guo et al., 2018). Most of LDs trackers utilized LDs’ hydrophobic neutral lipid cores, and cannot differentiate neutral lipid cores of LDs from intracellular other lipophilic compartments. They considered the exceptional interfacial architecture of LDs, and proposed a novel interface-targeting strategy for the first time. Through the lipophilic and electrostatic interaction, this amphiphilic tracker predominantly stained LDs by interface-targeting property in live HeLa cells. The co-localization experiments with the commercial LDs, nucleus, and mitochondria trackers demonstrated the high LD specificity of **5**. The OPEF signals of blue color are from Hoechst 33342 for the nucleus, green from **5** for LDs, and red from Mito Tracker Deep Red (MTDR) for mitochondria, respectively (Figure 2A). Through TPM imaging, it can be used to visualize the changes of LDs as well as their malfunctions in live hepatic tissues. This study can serve as an effective standard to monitor the changes in LDs and the interface-targeting strategy for designing organelle trackers with ultrahigh specificity.

Niu et al. developed a novel AIE based LDs tracker (**6**, NAP-CF_3_, Figure 1B) and described the specific visualization of LDs in live cells and tissues (Niu et al., 2018). They have synthesized AIE luminogens (AIEgens) that consist of a naphthalene core and four different electron-accepting groups. All these novel AIEgens showed good solid-state fluorescence quantum yield, large Stokes shift, and high TPA from 45 to 100 GM with 860 nm TP excitation. The use of an ultralow concentration of the fluorescent trackers could reduce the disruption of LD dynamics (Neef and Schultz, 2009; Kowada et al., 2015). Stained with an ultralow concentration (50–100 nM) in live HeLa cells, these AIEgens exhibited outstanding LD labeling ability with low background noise. Using **6** as the representative example, TP imaging analysis of LDs in live HeLa cells and liver tissues of mice at a depth of 70 μm (Figure 2B). **6** also showed remarkable photostability and biocompatibility. This research offers a useful method to ideally design and develop new AIEgens that are specific to LDs in live samples.

In 2020, Kim group reported six candidate compounds that are compatible with both OPM and TPM for monitoring of LDs in live hepatic cells and tissues (Cho et al., 2020). Among these compounds, **7** (Probe 3, Figure 1B) selectively stain LDs with a considerable TPA value (166 GM), probably due to the efficient ICT by the dimethylamino benzofuran donor and isophthalonitrile acceptor. It displayed the absorption and emission maxima at 444 nm and 513 nm, respectively, and a quantum yield of 0.98 in toluene. TPM imaging analysis using **7** revealed that the increase of LDs in response to ER stress or oleic acid in live cells and in mouse hepatic tissues. Importantly, **7** showed narrow absorption and emission spectra, so it is suitable for multicolor imaging. Therefore, this tracker is useful for monitoring of the interaction between LDs and other organelles as well as LDs-related cellular signaling in live samples. By co-staining with the commercial OP lysosomal tracker (Lyso Tracker Red), **7** allowed the reliable evaluation of steatosis and phospholipidosis in live hepatic cells after drug treatments. It might be a useful imaging tool for tracking the progress of LDs-related liver diseases as well as screening drugs for hepatotoxicity.

Later, Moon and Kim established an intravital TP imaging method using a small LDs tracker (**8**, SF-44, Figure 1B) and demonstrated the dynamic alteration of hepatic LDs (Moon and Kim, 2021). **8**, a TP tracker for LD imaging (Kim et al., 2015), was used to specifically stain LDs *in vivo* by intravenous injection. Within 10 min, labeled LDs in hepatocytes and the stained small molecular trackers were entirely cleared out of the cytoplasm after 4 h, which allowed repetitive intravital TPM imaging. Especially, they successfully visualized continuous accumulation of hepatic LDs after fasting for 48 h, probably due to the unnecessary influx of fatty acids distributed from adipocytes (Figure 2C). In addition, they found that the increases in the number of LDs with an average diameter of below 2 μm resulted in a markedly enlarged volume of hepatic LDs after fasting. The increased hepatic LDs after fasting were significantly diminished and the occupation of the hepatic LDs was restored to normal with refeeding for 24 h. This intravital TPM imaging method established in this study might be a highly advantageous imaging technique for future research into novel cellular mechanisms involved in the physiological and pathological dynamics of LDs.

In 2022, Han and co-workers synthesized a 5-(4-(diphenylamino)phenyl)thiophene-2-carbaldehyde as the TP LDs tracker (**9**, DTPC, Figure 1B), and applied it to monitor hepatic LDs in steatosis model (Han et al., 2022). They described the commercially viable LDs-specific TP tracker that possesses helpful characteristics, including a sharp absorption/emission band, a significant Stokes shift, and high brightness. **9** showed high polarity-dependency in various solvents, and obvious bathochromic shifts were observed as the polarity of the solvents increased. This revealed that the excited state of **9** has a larger dipole moment than the ground state. In various cell lines, including HeLa, A549, and L929, **9** exhibited bright fluorescent dots in all types of the cell lines, showing their universality in live cells. Moreover, the live hepatic tissues at a depth of 10 μm in steatosis model mice were successfully visualized in real-time with **9** (Figure 2D). In the near future, this polarity-sensitive, commercially feasible TP LDs tracker might be further extended to monitor the detailed origins and dynamics of LDs in live samples.

Most recently, Lee et al. developed a red-emissive TP tracker for LDs (**10**, LD1, Figure 1B), and visualized the dynamics of LDs in real-time live sample imaging (Lee et al., 2022). Although the conjugation length of **10** is shorter than that of **4-1**, **10** exhibited significant TPA values (150–610 GM) with the emission maxima at 520–640 nm. This might be attributed to the stronger electron donating efficiency of the cyclized diamine fused benzofuran moiety. When applied to TPM experiments in human hepatocyte cells and *in vivo* mouse hepatic tissues, **10** showed bright fluorescence specifically in LDs to assess the level of non-alcoholic fatty liver disease (Figure 2E). They did not only find that the hepatic sinusoids became narrower with the enlarged size of LDs but also monitored the moment of the fusion of LDs in hepatic diseased models. Most importantly, through the dual-color *in vivo* TPM imaging with a TP lysosome tracker, **10** might provide a promising drug screening method in pharmaceutical fields to evaluate the drug-induced liver injury inducing chemicals in advance. Later, intravital monitoring of the dynamic degradation of LDs in MS-275-treated mice was successfully accomplished by using **10** through TPM analysis (Lee et al., 2022). The multi-color imaging technique described in this research can serve as a powerful guideline to develop and apply novel fluorescent trackers for various organelles.

## Conclusion and outlook

Intensive studies have been conducted to monitor LDs in recent years to understand their complicated dynamics in cells. The fluorescence imaging technique is the most reliable method. However, the specific aspects of the biological functions and the related metabolisms of LDs in live samples remain challenging. Therefore, novel fluorescent trackers that have low self-absorption and background noise, high resolution, and photostability could be a powerful tool for studying the dynamics of LDs. Most recently, as described above, some of the TP fluorescent trackers for LDs have been developed with superior advantages to the OP trackers for LDs. Using TPM, these TP LDs trackers could provide high quality, real-time, deep tissue imaging in live cells and tissues. Furthermore, multi-color imaging with these TP LDs trackers and other organelle trackers might be useful for monitoring the organelle interactions in a certain physiological and pathological status. In bio-medical applications, these may serve as a diagnosis, prognosis, drug screening, or therapy agent of LDs-related diseases.

More consideration should be given to TP LDs trackers and their bio-medical applications in the future for the progressive advances in this important research topic. It is very important that LDs interact dynamically with other cellular organelles, especially in LD generation, cellular homeostasis, lipid and membrane synthesis, autophagy, and other processes. For example, Huang et al. (2022) visualized the LDs and lysosomes under lipophagy process, and Wang et al. (2020a) monitored the dynamic changes of LDs and mitochondria during oxidative stress. To get the fully reliable information about the complicated interactions of LDs with other organelles in various diseases, it is crucial to develop novel TP LDs trackers that cover the emission wavelengths from blue to deep red region in the future. The full-color TP LD trackers could be a valuable method to understand LDs-related phenomena in live samples.
